# Topological phase transitions without symmetry indication in NaZnSb$_{1-x}$Bi$_x$

**DOI:** 10.1038/s41598-022-26596-y

**Published:** 2022-12-21

**Authors:** Jaemo Jeong, Dongwook Kim, Youngkuk Kim

**Affiliations:** 1 Department of Physics, Sungkyunkwan University, Suwon, 16419 Korea; 2 Department of Materials Science and Engineering, University of Utah, Salt Lake City, UT 84112 USA

**Keywords:** Topological insulators, Topological insulators

## Abstract

We study topological phase transitions in tetragonal NaZnSb$_{1-x}$Bi$_x$, driven by the chemical composition *x*. Notably, we examine mirror Chern numbers that change without symmetry indicators. First-principles calculations are performed to show that NaZnSb$_{1-x}$Bi$_x$ experiences consecutive topological phase transitions, diagnosed by the strong ${\mathbb {Z}}_2$ topological index $\nu _{0}$ and two mirror Chern numbers $\mu _{x}$ and $\mu _{xy}$. As the chemical composition *x* increases, the topological invariants ($\mu _{x}\mu _{xy}\nu _{0}$) change from (000), (020), (220), to (111) at *x* = 0.15, 0.20, and 0.53, respectively. A simplified low-energy effective model is developed to examine the mirror Chern number changes, highlighting the central role of spectator Dirac fermions in avoiding symmetry indicators. Our findings suggest that NaZnSb$_{1-x}$Bi$_{x}$ can be an exciting testbed for exploring the interplay between the topology and symmetry.

## Introduction

Since the discovery of archetypal topological insulators protected by time-reversal symmetry^1,2^, many topological materials with potential applications have been discovered. According to the current topological materials databases^3–5^, out of the 24825 materials tested, 4321 are identified as topological (crystalline) insulators, and 10007 are identified as topological semimetals. Along with topological materials, diverse topological phases have been discovered, enriched by diverse symmetries such as translation^6–9^, inversion^10–14^, mirror^15^, rotation^16–19^, or glide mirror^20–23^, and with or without time-reversal symmetry^18,24–28^. Topological phases are also classified based on their order^29–32^, fragility^33^, delicacy^34^, obstructed^35–37^, and noncompact^38^ atomic insulators. They are applicable with outstanding results in various apparatuses, such as chemical^39,40^, electronic^27,41–44^, spintronic^45–49^, and quantum computer devices^50–53^.

The remarkable developments in topological band theory could be one of the fundamental reasons for the success in finding topological materials and phases^54,55^. Moreover, topological quantum chemistry, or equivalently, the symmetry-based indicator method^3,56,57^, has enabled efficient and high-throughput searches for topological materials. The symmetry indicator significantly simplifies the problem of identifying topological states for a given set of materials. Combined with the first-principles calculations based on density functional theory (DFT), band representations at high-symmetry momenta can efficiently indicate nontrivial band topology. Seemingly distinct topological phases are interconnected via symmetries of materials. Thus, inspecting the protecting symmetry has provided insights into determining the topological phases that share the protecting symmetries^56,58,59^.

Symmetry indicators are a robust scheme, but their limitations are apparent. Notably, they fail for a specific set of topological phases, referred to as fragile topological phases^33,60^, which have been a subject of intense study^61–65^. Moreover, the symmetry indicators intrinsically have a one-to-many nature^66^. Multiple stable topological phases exist for the same trivial indicator. Thus, the Berry phases and Wilson-loop calculations should be employed to determine the stable topological phase. This one-to-many nature allows for a disjointed distinction between the topological phase transitions with and without symmetry indicators. In this study, we examine a class of topological phase transitions that cannot be found in the symmetry indicators. These symmetry-uncaught topological phase transitions can occur because of the lack of symmetry to discern the topological phase transition in terms of symmetry representation^67,68^. However, the detailed process of topological phase transitions to avoid symmetry indication remains unexplored.

In this paper, we present a case study of a stable topological phase transition that occurs without symmetry indications. We perform first-principles calculations to study the topological phase transitions of NaZnSb$_{1-x}$Bi$_{x}$ in the presence of time-reversal symmetry driven by the chemical composition *x*, diagnosed by two mirror Chern numbers $\mu _{x}$ and $\mu _{xy}$ and the strong ${\mathbb {Z}}_{2}$ topological index $\nu _{0}$. $(\mu _{x}\mu _{xy}\nu _{0})$ changes from (000), (020), (220), to (111) at *x* = 0.15, 0.20, and 0.53, respectively. Among these, the topological phase transitions from (000) to (020) and from (020) to (220) occur within the same (trivial) symmetry indicators, thereby uncaught from the symmetry indicators. We build a simplified effective model to demonstrate a mirror Chern number change between the bands with the same symmetry representation, forbidding symmetry indication. We find that symmetry plays a role in the phase transition by providing a constraint on the positions of Dirac fermions and spectator Dirac fermions^69–71^ in momentum space.

### Crystal structure and symmetries

**Figure 1:**
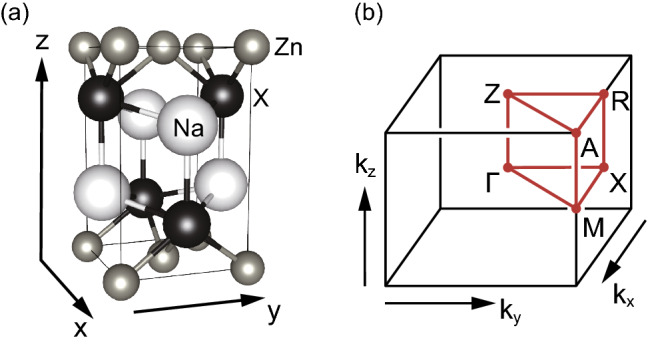
(**a**) Crystal structure of matlockite-type NaZn*X* (*X* = Sb and Bi) in space group *P*4/nmm (# 129). Na, Zn, and *X* atoms are colored by white, grey, and black, respectively. The unit cell is represented by a solid (black) box. (**b**) Corresponding tetragonal first Brillouin zone. High-symmetry momenta are colored red.

Figure 1a shows the crystal structure of NaZn*X* ($X=$ Bi, Sb) in the space group *P*4/nmm (#129). The system comprises Na-*X* staggered-square sublattices and Zn planar square sublattices, placed between the Na-X bilayers. The *P*4/nmm space group has three generators - two screw rotations $\{C_{4z}\vert \tfrac{1}{2}\tfrac{1}{2}0\}$ and $\{C_{2x}\vert \tfrac{1}{2}\tfrac{1}{2}0\}$ and spatial inversion $\{{\mathcal {P}}\vert \tfrac{1}{2}\tfrac{1}{2}0\}$. $C_{4z}$ and $C_{2x}$ are fourfold and twofold rotations about the ${\varvec{z}}$-axis and ${\varvec{x}}$-axis, respectively (Fig. 1a), and $\{\, g \, \vert \tfrac{1}{2}\tfrac{1}{2}0\}$ ($g = C_{4z}, C_{2x}$, or ${\mathcal {P}}$) is a symmetry operator *g* followed by a fractional translation by half of the primitive unit vectors along the ${\varvec{x}}$- and ${\varvec{y}}$-directions. Notably, there exist *x*-mirror $M_{x}$ and *xy*-glide $G_{xy} = \{M_{xy}\vert \tfrac{1}{2}\tfrac{1}{2}0\}$, which will be employed to evaluate the mirror Chern numbers $\mu _{x}$ and $\mu _{xy}$, respectively. In addition, the system preserves time-reversal symmetry ${\mathcal {T}}$, enabling the ${\mathbb {Z}}_{2}$ topological insulator phase. The first Brillouin zone and the corresponding high-symmetry momenta are shown in Fig. 1b. Moreover, NaZnSb is an existing material^72–75^, whereas NaZnBi is yet to be synthesized.

### DFT bands

**Figure 2:**
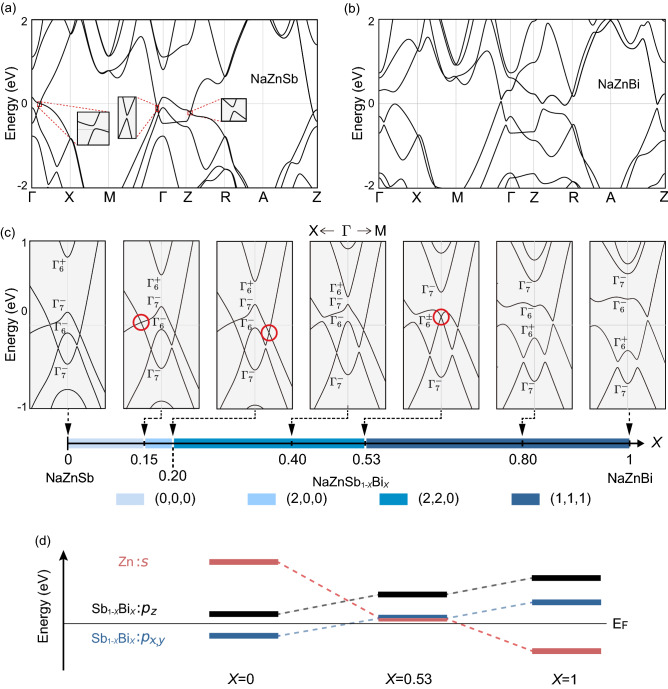
(**a**) First-principles bands of NaZnSb (*x* = 0) with spin-orbit coupling. The red rectangles are magnified in the guided grey boxes, showing a direct bandgap. (**b**) DFT bands of NaZnBi (*x* = 1). (**c**) DFT bands of NaZnSb$_{1-x}$Bi$_x$ for various *x* and topological phase diagram in *x*-space. The bands are drawn near the $\Gamma$ point along with the *X* and *M* directions, with the corresponding chemical composition *x* being indicated in the topological phase diagram. The red circles in the bands highlight the massless (zero-gap) Dirac points, which appear at the topological phase boundaries. Domains in different colors indicate distinct topological phases. (**d**) Schematic diagram of the orbital characters at $\Gamma$ as a function of *x*. The Zn-*s* and Sb$_{1-x}$Bi$_x$-$p_x$ and $p_y$ orbitals are inverted at *x* = 0.53, responsible for the change of the strong ${\mathbb {Z}}_2$ index.

Figure 2 shows the first-principles electronic energy bands of NaZnSb$_{1-x}$Bi$_x$ calculated for various chemical compositions *x* using virtual crystal approximation^76,77^. A close inspection reveals that a direct bandgap exists throughout the BZ for any $x\in [0,1]$ except for the cases where $x=0.15$, $x=0.20$, and $x=0.53$. In these fine-tuned compositions, the bandgap between the conduction and valence bands vanishes such that it can form a fourfold-degenerate band crossing with linear dispersion, which is dubbed by the Dirac point. Specifically, for the case where $x=0.15$ and $x=0.20$, the Dirac point appears on the $\Gamma -X$ and $\Gamma -M$ lines, respectively, contained in the $M_{x}$ ($G_{xy}$) invariant $k_{x}=0$ ($k_{x}=-k_{y}$) plane. However, for $x=0.53$, the Dirac point appears at the time-reversal invariant $\Gamma$ point and mediates the band inversion between the $\Gamma _6^+$ and $\Gamma _6^-$ states, as shown in Fig. 2c. The $\Gamma _6^+$ and $\Gamma _6^-$ states mainly comprise the Zn *s* and Sb$_{1-x}$Bi$_x$ $p_x$ and $p_y$ orbitals, respectively, as shown in Fig. 2d For any $x \in [0,1]$ other than these critical values, the conduction and valence bands are well separated by a direct bandgap, enabling the evaluation of the topological insulating phase from the occupied bands.

### Topological phases

The Dirac points accompany a topological phase transition. Using the Wilson loop calculations^18,79,80^, we enumerate two mirror Chern numbers $\mu _{x}$ and $\mu _{xy}$ associated with the $M_x$-mirror and $G_{xy}$-glide on the corresponding invariant planes at $k_x = 0$ and $k_x = -k_y$, respectively. (See Supplementary Information for the detailed calculations of the mirror Chern numbers). In addition, the three-dimensional strong ${\mathbb {Z}}_2$ topological invariant $\nu _{0}$ is calculated using the parity eigenvalues of the occupied bands at eight time-reversal invariant momenta^12^. As summarized in the bottom panel of Fig. 2c, we identify the topological phases characterized by ($\mu _{x},\mu _{xy},\nu _{0}$) = (0,0,0) for $0 \le x < 0.15$, (2,0,0) for $0.15< x < 0.20$, (2,2,0) for $0.20< x < 0.53$, and (1,1,1) for $0.53 < x \le 1$. We note that the calculated ${\mathbb {Z}}_2$ indices for $x=1$ are in good agreement with the previous result^81^. Correspondingly, topological phase transitions at $x=0.15$, $x=0.20$, and $x=0.53$ occur owing to the changes in the mirror Chern numbers $\mu _{x}$ and $\mu _{xy}$ and the strong ${\mathbb {Z}}_{2}$ topological index, respectively.

For completeness, we evaluate the other possible topological crystalline phases allowed in NaZnSb$_{1-x}$Bi$_x$. First, the three-dimensional weak topological insulator phases, characterized by the three weak ${\mathbb {Z}}_2$ indices $(\nu _1\nu _2\nu _3)$, are turned out to be all trivial $(\nu _1\nu _2\nu _3)=(0,0,0)$ for all gapped phase. In addition, the ${\mathbb {Z}}_{4}$ index associated with $\mathcal{P}\mathcal{T}$ symmetry^82^, denoted by $\nu _{4}$ is calculated as identical to the ${\mathbb {Z}}_{2}$ index $\nu _{0}$. Thus, $\nu _{4}=0$ for $x < 0.53$ and $\nu _{4}=1$ for $x >0.53$. Finally, the remaining topological indices are listed in Table 1. Despite the variety, the whole topological crystalline insulator phases are unambiguously determined by the weak indices and the ${\mathbb {Z}}_{4}$ index $(\nu _{1},\nu _{2}\nu _{3}\nu _{4})$ along with the two mirror Chern numbers $\mu _{x}$ and $\mu _{xy}$^66^. The mirror Chern number $\mu _{z}$ is associated with the glide $g_{z} = \{M_{z}\vert \tfrac{1}{2}\tfrac{1}{2}0\}$ symmetry. The $g_{z}$-invariant plane $k_{z}=0$ hosts four Dirac points at the critical composition $x=0.15$ and $x=0.20$. The mirror Chern number remains trivial, $\mu _{z}=0$ for $x<0.53$, which is consistent with the symmetry constraint dictated by $\nu _{4}=0$^66^.

**Figure 3:**
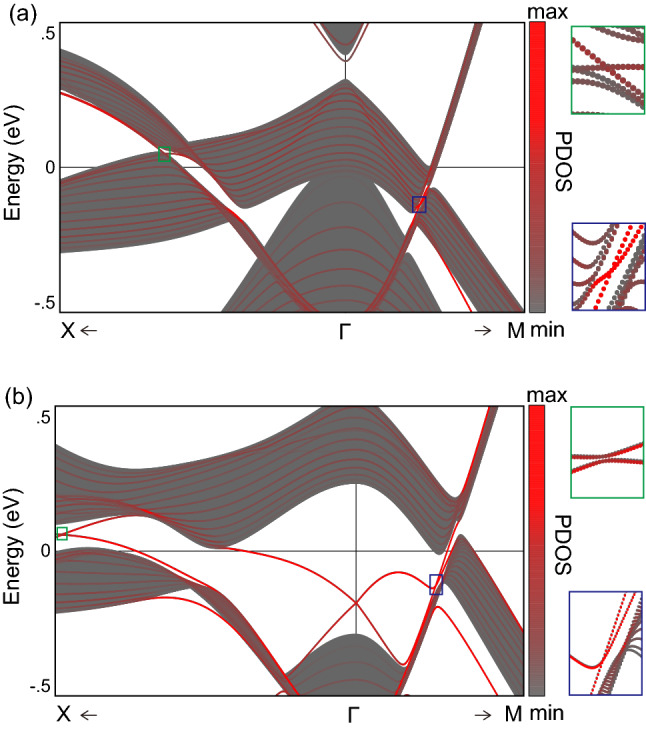
(001) surface energy spectra of NaZnSb$_{1-x}$Bi$_{x}$ at (**a**) *x* = 0.31 and (**b**) *x* = 1.00. The discrete energy spectra calculated from a slab geometry are overlapped with the continuous grey bulk projected spectra. The strength of the surface projection is indicated in red; the more substantial the red color, the stronger the localization at the surface. The right panels show the magnified views of the boxed regions in the left panels. The band crossing (anticrossing) is shown in the magnified views for the case of $x=0.31$ (*x*=1.00).

**Table 1:** Possible topological crystalline phases corresponding to the trivial $3{\mathbb {Z}}_2\times {\mathbb {Z}}_4$ symmetry indicators ($\nu _{1}\nu _{2}\nu _{3}\nu _{4}$) = (0,0,0,0) in space group #129.

Space group #129 : P4/nmm								
${\mathbb {Z}}_{2,2,2,4}$	$\mu _{x,0(\pi )}$	$\mu _{xy}$	$g_{z}$	$g_{xy}$	$c_{2z}$	$c_{2x{\overline{y}}}$	$c_{4z}$	$s_{2x}$
0000	0 (0)	0	0	0	0	0	0	0
0000	2 (0)	0	0	0	0	0	1	1
0000	0 (0)	2	0	1	0	1	1	0
0000	2 (0)	2	0	1	0	1	0	1

$\mu _{i}$ and $g_{i}$ and $c_{i}$, and $s_{i}$ are the mirror and glide and rotation and screw-resolved topological invariants about the *i*-invariant plane and the *i*-axis, respectively ($i=x, xy, \cdots$). We have applied the results of Ref.^66^.

### Topological surface states

The nontrivial topology found in NaZnSb$_{1-x}$Bi$_{x}$ for $x>0.15$ is demonstrated by explicit calculations of topological surface states. We prepared a slab geometry of NaZnSb$_{1-x}$Bi$_{x}$ comprising 15 unit cells along the [001]-direction with open boundary conditions imposed on the (001) surface. Figure 3 shows the computed surface states for (a) *x* = 0.31 and (b) *x* = 1.00, where $(\mu _{x}\mu _{xy}\nu _{0})=(2,2,0)$ and (1,1,1), respectively. When *x* = 0.31, the two surface Dirac points occur because of $\mu _{x}=2$ and $\mu _{xy}=2$ along the high-symmetry $\Gamma -X$ and $\Gamma -M$ lines of the surface BZ, respectively, where the nontrivial mirror planes are projected (Fig. 3a). For the case of $x=1.00$, on the other hand, the strong topological insulator phase is hosted ($\nu _{0}=1$), leading to the formation of a two-dimensional surface Dirac point occurring at the surface $\Gamma$ point (Fig. 3b). The calculated surface spectra agree well with the topological phases diagnosed from the bulk topological invariants.

### Symmetry indicators

After identifying the topological phases of NaZnSb$_{1-x}$Bi$_x$, we directly evaluate the symmetry indicators, and show that the symmetry indicators proposed in this space group fail to capture the topological phase transitions at $x=0.15$ and $x=20$. According to Ref.^66^, NaZnSb$_{1-x}$Bi$_x$ in space group #129 contains a set of $3{\mathbb {Z}}_2\times {\mathbb {Z}}_4$ symmetry indicators $(\nu _{1}\nu _{2}\nu _{3}\nu _{4})$. As introduced earlier, the first three indices $\nu _{i=1,2,3}$ are the three-dimensional weak ${\mathbb {Z}}_2$ topological indices, evaluated from the parity eigenvalues of the occupied bands^12^ and the last index $\nu _4$ is the ${\mathcal {P}}{\mathcal {T}}$ symmetric topological invariant, evaluated from $\nu _4 \equiv \sum _{\Gamma _i \in \mathrm TRIM} \tfrac{n^-_{\Gamma _i} - n^+_{\Gamma _i}}{2}$ (mod 4), where $n^{+(-)}_{\Gamma _i}$ is the number of even- (odd-) parity valence bands at a time-reversal invariant momentum $\Gamma _i$^82^. From the first-principles calculations of symmetry representations, we obtain the symmetry indicators $(\nu _{1}\nu _{2}\nu _{3}\nu _{4})=(0000)$ for $x < 0.53$ and (0001) for $x > 0.53$. Thus, the change in the strong index at $x = 0.53$ is captured by the symmetry indicators, but those at $x=0.15$ and $x=0.20$ are unseen. The absence of a symmetry indication can be attributed to the symmetry representations of the bands. Because the topological phase changes via the formation of the Dirac points that reside off the high-symmetry momenta, the symmetry representations of the bands remain the same immediately before and after the Dirac point. Therefore, the failure of the symmetry indicators is inevitable, as evaluated from the symmetry representations.

We explain the failure of symmetry indicators as being due to the so-called symmetry-allowed nature of the Chern numbers. Unlike the symmetry-protected topological phases, the Chern number characterizes a so-called symmetry-forbidden phase, in which symmetries play a role in giving rise to a constraint instead of protection. As shown by Song et al.^66^, there are four varieties for a given symmetry indicator in space group #129 (See Table 1). The varieties arise from the two possibilities of the two mirror Chern numbers, that is, $\mu _{i}=0,2$ for $i=x, xy$. which are under the symmetry constraints for the two-fold $C_{2i}$ rotation^17^

1 $$\begin{aligned} e^{i \pi \mu _{i}} = \prod _{n \in \mathrm {occ.}} (-1)^F\prod _{\Gamma _a \in \textrm{RIM}}\theta _n(\Gamma _a), \end{aligned}$$

where $\theta _n (\Gamma _a) = e^{i (2J_n^{a} +F)\pi /2}$, $J_n^{a}$ is an eigenvalue of the $C_{2i}$ rotation for the *n*-th occupied band at a rotation-invariant momenta (RIM) $\Gamma _{a}$ contained in the mirror-invariant plane, and $F = 1 (0)$ for a spinful (spinless) system. Therefore, the Chern number can be changed by determining $\Delta {\mathcal {C}}$ from

2 $$\begin{aligned} e^{i\pi \Delta {\mathcal {C}}} = 1, \end{aligned}$$

or equivalently,

3 $$\begin{aligned} \Delta {\mathcal {C}} = 0 ~(\text {mod~} 2) \end{aligned}$$

when the $J_{n}^{a}$ remains the same before and after the variations in Chern number. Thus, $\mu _{i} = 0$ and $\mu _{i} = 2$ are symmetry-allowed, enabling the varieties of topological phases under the same symmetry structure.

### Mirror-specific four-band model

We further resolve the role of symmetry in the change in mirror Chern numbers by constructing an effective Hamiltonian. Let us begin with a generic $4\times 4$ Hamiltonian

4 $$\begin{aligned} {\mathcal {H}}({\varvec{k}}) = \sum _{i,j = x,y,z} h_{ij} ({\varvec{k}}) \tau _i \sigma _j, \end{aligned}$$

where $\tau _{x,y,z}$ and $\sigma _{x,y,z}$ are the Pauli matrices describing the orbitals and spins, respectively. The $D_{2h}$ point-group symmetries are distilled from the DFT bands responsible for the mirror Chern number change (See Supplementary Information for the detailed derivation of the effective model). This leads to the symmetry representations: ${\mathcal {T}} = i\sigma _z {\mathcal {K}}$, $M_{x,y,z} = i\sigma _{x,y,z}$, and $\mathcal P = {\mathcal {I}}_{4\times 4}$. Here, ${\mathcal {K}}$ is the complex conjugation. Under the symmetry constraints

5 $$\begin{aligned} {\mathcal {H}}({\hat{O}}_g {\varvec{k}}) = U_g^\dag {\mathcal {H}}({\varvec{q}} ) U_g, \end{aligned}$$

where $U_g$ and ${\hat{O}}_g$ are the representation for the symmetry operator *g* in matrix and momentum spaces, respectively, the effective Hamiltonian on the mirror-invariant plane $k_z = 0$ is obtained as

6 $$\begin{aligned} {\mathcal {H}}({\varvec{k}}) = A(k_x,k_y) \tau _x + B(k_x,k_y) \tau _z + C(k_x,k_y) \tau _y \sigma _z, \end{aligned}$$

where $A(k_x,k_y) \equiv a_0+a_1 k_x^2+a_2 k_y^2$, $B(k_x,k_y) \equiv b_0+b_1 k_x^2+b_2 k_y^2$, and $C(k_x,k_y) \equiv c_2 k_x k_y$ to the quadratic order in ${\varvec{k}} = (k_x,k_y)$. The corresponding energy bands are given by

7 $$\begin{aligned} E_{\pm } ({\varvec{k}}) = \pm \sqrt{A(k_x,k_y)^2 + B(k_x,k_y)^2 + C(k_x,k_y)^2}, \end{aligned}$$

for each mirror-sector $\sigma _z = \pm 1$. The parameters $a_i, b_i$, and $c_2$ (*i* = 0, 1,and 2) can be fine-tuned to critical points, where $A = B = C = 0$. These conditions lead to a bandgap crossing $E_{+} = E_{-}$ at ${\varvec{k}} = (\pm \sqrt{-a_0/a_1},0)$ or ${\varvec{k}} = (0,\pm \sqrt{-a_0/a_2})$ (We note that $c_2 = 0$ can also close band gap, but the mirror Chern number remains the same via the gap closer. See the Supplemental Information for the detailed calculations.)

**Figure 4:**
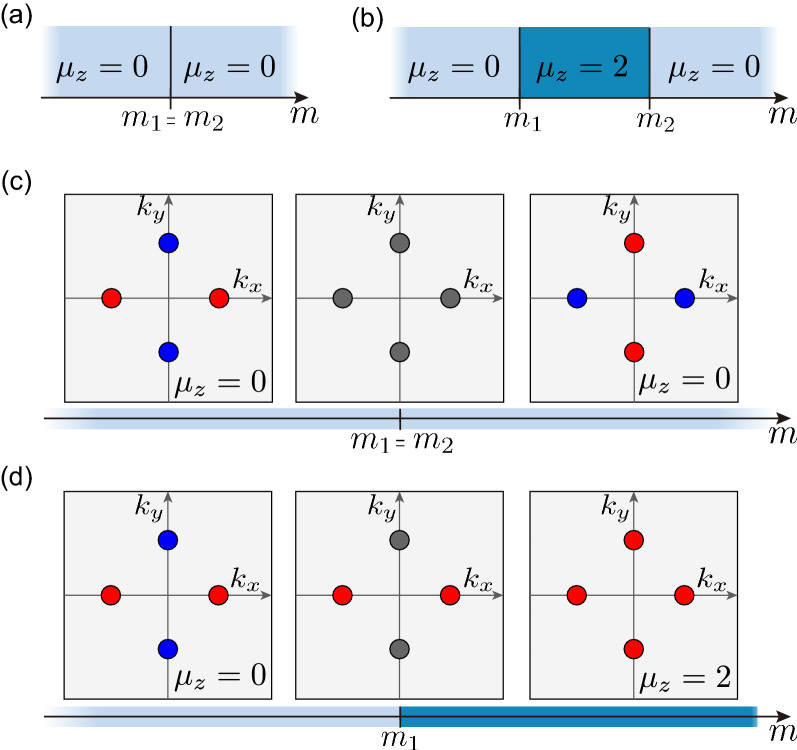
Schematic phase diagrams for a topological crystalline phase transition diagnosed by mirror Chern number $\mu _{z}$. (**a**) Systems with $C_{4z}$. The Dirac and spectator Dirac points that close the band gap simultaneously at $m = m_1 = m_2$ result in the same topological crystalline phases with the mirror Chern number ${\mathcal {C}}_M = 0$. (**b**) Systems without $C_{4z}$. The Dirac and spectator Dirac points that close the band gap independently at $m = m_1$ and $m = m_2 \ne m_1$ can mediate a topological phase transition from $\mu _{z} = 0$ to $\mu _{z} = 2$. (**c**–**d**) Mass inversion of Dirac fermions in the mirror-invariant plane of momentum space: (**c**) with $C_{4z}$ and (**d**) without $C_{4z}$. The red and blue circles represent the massive Dirac and the massive spectator Dirac fermions, respectively. The grey circles indicate the massless Dirac fermions.

The Chern number that characterizes the occupied bands $E_- ({\varvec{k}})$ for each mirror-sectors $M_z = \pm i$ is determined by

8 $$\begin{aligned} {\mathcal {C}}_{\pm i} = \pm \left\{ \textrm{sgn} \left[ c_2 \left( \frac{a_0}{a_1} - \frac{b_0}{b_1}\right) \right] - \textrm{sgn}\left[ c_2\left( \frac{a_0}{a_2} - \frac{b_0}{b_2}\right) \right] \right\} , \end{aligned}$$

from which the mirror Chern number $\mu _z$^15,16^ can be obtained as

9 $$\begin{aligned} \mu _{z} \equiv \frac{1}{2} ({\mathcal {C}}_{+i} - {\mathcal {C}}_{-i}) = \textrm{sgn}\left[ c_2 \left( \frac{a_0}{b_0} - \frac{a_2}{b_2}\right) \right] - \textrm{sgn}\left[ c_2\left( \frac{a_0}{b_0} - \frac{a_1}{b_1}\right) \right] . \end{aligned}$$

The nontrivial (trivial) topological crystalline phase indexed by $\mu _{z}=2$ (=0) occurs when $\left( a_0 b_2 - a_2 b_0\right) \left( a_0 b_1 - a_1 b_0\right) <0(>0)$. This equation directly shows that the bandgap crossings define the topological phase transitions between $\mu _z=2$ and $\mu _z=0$.

As illustrated in Fig. 4, the results of the effective model provide essential insight into the role of symmetries. The *n*-fold rotational symmetry generates *n* symmetry-related Dirac fermions whose mass is flipped simultaneously during the phase transition. This leads to variations in the Chern number with *n*. We believe that the fraction of *n* can only be changed when the symmetry is implicitly broken at the representation level, which can be deduced from the symmetry indicators. It is interesting to note the role of the spectator Dirac fermions^69,69–71^, which refers to the massive Dirac fermions without mass inversion during the transition. Upon restoring a higher-rotational symmetry, such as $C_{4z}$, the topological phase transition becomes trivialized by enforcing the participation of the spectator Dirac fermions. In our case, the $C_{4z}$-symmetry enforces $a_1 = a_2$ and $b_1 = b_2$, and thus, all the massive Dirac fermions invert the mass simultaneously to nullify the mirror Chern number change. This conforms to the symmetry constraint given by $C_{4z}$ to the mirror Chern number. It can only change integers that are multiples of four, forbidding two. We believe that this occurs in NaZnSb$_{1-x}$Bi$_{x}$ at $x=0.15$ and $x=0.20$, where four Dirac points occur on the $G_{z}$-invariant $k_{z}=0$ plane without changing the mirror Chern number $\mu _{z}=0$.

## Conclusions

We have performed a first-principles study on the topological phases of NaZnSb$_{1-x}$Bi$_x$ driven by the chemical composition *x*. We have established the topological phase diagram in *x*-space using symmetry indicators, two mirror Chern numbers, and the ${\mathbb {Z}}_{2}$ strong topological index. The phase boundaries are determined to be *x*=0.17, 0.20, and 0.53. We focused on analyzing the first two topological phase transitions, which changed the mirror Chern numbers without symmetry indications. The absence of a symmetry indication is attributed to the intrinsic nature of the Chern numbers. In general, the Chern number can jump by a factor of *n* without being caught by the $C_{n}$-symmetry, which can be fulfilled by hosting *n* massless Dirac fermions that mediate the change in the Chern number.

Our results are scientifically innovative in three aspects. First, the study provides insights into topological phase transitions, uncovering the close interplay between symmetry and topology. Second, we highlight the one-to-many nature of symmetry indicators, suggesting that materials identified as trivial in topology via symmetry inputs can be nontrivial. This may provide opportunities for finding topological materials. Finally, NaZnSb$_{1-x}$Bi$_{x}$ in the tetragonal phase is such an archetypal example that suggests a rich playground for exploring topological phenomena. For example, we believe that the Fermi surface topology as a function of doping concentration and chemical potential will be an interesting future study in the NaZnSb$_{1-x}$Bi$_x$ systems.

## Methods

We performed first-principles calculations based on density functional theory (DFT) as implemented in Quantum Espresso package^83^. We used the Perdew-Burke-Ernzerhof (PBE) type general gradient approximation for exchange-correlation functional^84^. The Opium package is used to construct norm-conserving, optimized, designed non-local, and fully-relativistic pseudopotentials for the Na, Zn, Bi, and Zn atoms^85,86^. The atomic structures are fully relaxed within the force criterion of 10$^{-7}$ eV/Å. The wave functions are expanded on a plane-wave basis with an energy cutoff of 680 eV. The atomic structures are fully relaxed within the force threshold of 10$^{-5}$ eV/Å. The 8$\times$8$\times$8 sampling of the ${\varvec{k}}$-point grid is used based on the Monhorst-Pack scheme^87^. We have tested that this ${\varvec{k}}$-point grid is dense enough to achieve the self-consistent charge density and total energy convergence. Atomic substitution from Sb to Bi as a function of chemical composition *x* is mimicked by virtual crystal approximation^76,77^. The lattice parameters of the tetragonal unit cell are calculated as *a* = 4.39 Å, *c* = 7.36 Å for the case of *X* = Sb and *a* = 4.54 Å, *c* = 7.55 Å for the case of *X* = Bi. The unitcell comprises two formula units with six atoms Na1, Na2, Zn1, Zn2, *X*1, and *X*2 located at (0.25*a*, 0.25*a*, 0.16*c*), (0.75*a*, 0.75*a*, 0.84*c*), (0.75*a*, 0.25*a*, 0.5*c*), (0.25*a*, 0.75*a*, 0.5*c*), (0.75*a*, 0.75*a*, 0.27*c*), and (0.25*a*, 0.25*a*, 0.73*c*), respectively. The mirror Chern numbers^15,16^ were calculated using the mirror-specified Wilson loop calculations^18,78–80^. The Wilson Hamiltonians are generated by the Soluyanov and Vanderbilt method^88^, using the Pw2wan utility in Wannier90 code^89–93^.

## Supplementary Information

Supplementary Information.

## Data Availability

The datasets generated from the current study are available from the corresponding author upon reasonable request.

## References

[1] Hasan MZ, Kane CL (2010). Colloquium: Topological insulators. Rev. Mod. Phys..

[2] Qi X-L, Zhang S-C (2011). Topological insulators and superconductors. Rev. Mod. Phys..

[3] Bradlyn B, Elcoro L, Cano J, Vergniory M, Wang Z, Felser C, Aroyo MI, Bernevig BA (2017). Topological quantum chemistry. Nature.

[4] Vergniory M, Elcoro L, Felser C, Regnault N, Bernevig BA, Wang Z (2019). A complete catalogue of high-quality topological materials. Nature.

[5] Vergniory, M. G., Wieder, B. J., Elcoro, L., Parkin, S. S., Felser, C., Bernevig, B. A. & Regnault, N. All topological bands of all stoichiometric materials. arXiv preprint arXiv:2105.09954 (2021). 10.1126/science.abg9094 35587971

[6] Fu L, Kane CL, Mele EJ (2007). Topological insulators in three dimensions. Phys. Rev. Lett..

[7] Moore JE, Balents L (2007). Topological invariants of time-reversal-invariant band structures. Phys. Rev. B.

[8] Cheng M, Zaletel M, Barkeshli M, Vishwanath A, Bonderson P (2016). Translational symmetry and microscopic constraints on symmetry-enriched topological phases: A view from the surface. Phys. Rev. X.

[9] Song H, Huang S-J, Fu L, Hermele M (2017). Topological phases protected by point group symmetry. Phys. Rev. X.

[10] Turner AM, Zhang Y, Vishwanath A (2010). Entanglement and inversion symmetry in topological insulators. Phys. Rev. B.

[11] Hughes TL, Prodan E, Bernevig BA (2011). Inversion-symmetric topological insulators. Phys. Rev. B.

[12] Fu L, Kane CL (2007). Topological insulators with inversion symmetry. Phys. Rev. B.

[13] Ahn J, Kim D, Kim Y, Yang B-J (2018). Band topology and linking structure of nodal line semimetals with Z^2^ monopole charges. Phys. Rev. Lett..

[14] Jeon S, Kim Y (2022). Two-dimensional weak topological insulators in inversion-symmetric crystals. Phys. Rev. B.

[15] Teo JCY, Fu L, Kane CL (2008). Surface states and topological invariants in three-dimensional topological insulators: Application to $${\text{bi}}_{{1 - x}} {\text{sb}}_{x}$$. Phys. Rev. B.

[16] Fu L (2011). Topological crystalline insulators. Phys. Rev. Lett..

[17] Fang C, Gilbert MJ, Dai X, Bernevig BA (2012). Multi-Weyl topological semimetals stabilized by point group symmetry. Phys. Rev. Lett..

[18] Alexandradinata A, Fang C, Gilbert MJ, Bernevig BA (2014). Spin-orbit-free topological insulators without time-reversal symmetry. Phys. Rev. Lett..

[19] Fang C , Fu L (2019). New classes of topological crystalline insulators having surface rotation anomaly. Sci. Adv..

[20] Young SM, Kane CL (2015). Dirac semimetals in two dimensions. Phys. Rev. Lett..

[21] Fang C, Fu L (2015). New classes of three-dimensional topological crystalline insulators: Nonsymmorphic and magnetic. Phys. Rev. B.

[22] Wang Z, Alexandradinata A, Cava RJ, Bernevig BA (2016). Hourglass fermions. Nature.

[23] Wieder BJ, Bradlyn B, Wang Z, Cano J, Kim Y, Kim H-SD, Rappe AM, Kane C, Bernevig BA (2018). Wallpaper fermions and the nonsymmorphic Dirac insulator. Science.

[24] Li R, Wang J, Qi X-L, Zhang S-C (2010). Dynamical axion field in topological magnetic insulators. Nat. Phys..

[25] Burkov AA, Hook MD, Balents L (2011). Topological nodal semimetals. Phys. Rev. B.

[26] Bonderson P, Nayak C, Qi X-L (2013). A time-reversal invariant topological phase at the surface of a 3d topological insulator. J. Stat. Mech. Theory Exp..

[27] Tokura Y, Yasuda K, Tsukazaki A (2019). Magnetic topological insulators. Nat. Rev. Phys..

[28] Elcoro L, Wieder BJ, Song Z, Xu Y, Bradlyn B, Bernevig BA (2021). Magnetic topological quantum chemistry. Nat. Commun..

[29] Benalcazar WA, Bernevig BA, Hughes TL (2017). Quantized electric multipole insulators. Science.

[30] Schindler F, Cook AM, Vergniory MG, Wang Z, Parkin SS, Bernevig BA, Neupert T (2018). Higher-order topological insulators. Science advances.

[31] Khalaf E (2018). Higher-order topological insulators and superconductors protected by inversion symmetry. Phys. Rev. B.

[32] Călugăru D, Juričić V, Roy B (2019). Higher-order topological phases: A general principle of construction. Phys. Rev. B.

[33] Po HC, Watanabe H, Vishwanath A (2018). Fragile topology and Wannier obstructions. Phys. Rev. Lett..

[34] Nelson A, Neupert T, Bzdušek TCV, Alexandradinata A (2021). Multicellularity of delicate topological insulators. Phys. Rev. Lett..

[35] Cano J, Bradlyn B, Wang Z, Elcoro L, Vergniory MG, Felser C, Aroyo MI, Bernevig BA (2018). Building blocks of topological quantum chemistry: Elementary band representations. Phys. Rev. B.

[36] Xu, Y., Elcoro, L., Song, Z.-D., Vergniory, M. G., Felser, C., Parkin, S. S. P., Regnault, N., Mañes, J. L. & Bernevig, B. A. Filling-enforced obstructed atomic insulators. arXiv:2106.10276 (2021).

[37] Cano J, Elcoro L, Aroyo MI, Bernevig BA, Bradlyn B (2022). Topology invisible to eigenvalues in obstructed atomic insulators. Phys. Rev. B.

[38] Schindler F, Bernevig BA (2021). Noncompact atomic insulators. Phys. Rev. B.

[39] Sattigeri, R. M., Jha, P. K., Śpiewak, P. & Kurzydłowski, K. J. Two dimensional limgas; a novel topological quantum catalyst for hydrogen evolution reaction. arXiv preprint arXiv:2204.08926 (2022)

[40] Kim D, Liu F (2022). Topological alloy engineering and locally linearized gap dependence on concentration. Phys. Rev. B.

[41] Sacépé B, Oostinga JB, Li J, Ubaldini A, Couto NJ, Giannini E, Morpurgo AF (2011). Gate-tuned normal and superconducting transport at the surface of a topological insulator. Nat. Commun..

[42] Steinberg H, Laloë J-B, Fatemi V, Moodera JS, Jarillo-Herrero P (2011). Electrically tunable surface-to-bulk coherent coupling in topological insulator thin films. Phys. Rev. B.

[43] Kong D, Chen Y, Cha JJ, Zhang Q, Analytis JG, Lai K, Liu Z, Hong SS, Koski KJ, Mo S-K (2011). Ambipolar field effect in the ternary topological insulator (bixsb1-x) 2te3 by composition tuning. Nat. Nanotechnol..

[44] Dhori BR, Sattigeri RM, Jha PK, Kurzydlowski D, Chakraborty B (2022). A first-principles investigation of pressure induced topological phase transitions in half-Heusler AgSrBi. Mater. Adv..

[45] Hsieh D, Qian D, Wray L, Xia Y, Hor YS, Cava RJ, Hasan MZ (2008). A topological Dirac insulator in a quantum spin hall phase. Nature.

[46] Zhang H, Liu C-X, Qi X-L, Dai X, Fang Z, Zhang S-C (2009). Topological insulators in bi2se3, bi2te3 and sb2te3 with a single Dirac cone on the surface. Nat. Phys..

[47] Chen YL, Analytis JG, Chu J-H, Liu ZK, Mo S-K, Qi XL, Zhang HJ, Lu DH, Dai X, Fang Z, Zhang SC, Fisher IR, Hussain Z, Shen Z-X (2009). Experimental realization of a three-dimensional topological insulator Bi_2_Te_3_. Science.

[48] Garate I, Franz M (2010). Inverse spin-galvanic effect in the interface between a topological insulator and a ferromagnet. Phys. Rev. Lett..

[49] He M, Sun H, He QL (2019). Topological insulator: Spintronics and quantum computations. Front. Phys..

[50] Freedman MH, Larsen M, Wang Z (2002). A modular functor which is universal¶ for quantum computation. Commun. Math. Phys..

[51] Das Sarma S, Nayak C, Tewari S (2006). Proposal to stabilize and detect half-quantum vortices in strontium ruthenate thin films: Non-abelian braiding statistics of vortices in a $${p}_{x}+i{p}_{y}$$ superconductor. Phys. Rev. B.

[52] Nayak C , Simon SH, Stern A, Freedman M, Das Sarma S (2008). Non-abelian anyons and topological quantum computation. Rev. Mod. Phys..

[53] Checkelsky JG, Ye J, Onose Y, Iwasa Y, Tokura Y (2012). Dirac-fermion-mediated ferromagnetism in a topological insulator. Nat. Phys..

[54] Kane CL (2013). Topological band theory and the Z$$_2$$ invariant. Contemporary Concepts of Condensed Matter Science.

[55] Bansil A, Lin H, Das T (2016). Colloquium: Topological band theory. Rev. Mod. Phys..

[56] Kruthoff J, de Boer J, van Wezel J, Kane CL, Slager R-J (2017). Topological classification of crystalline insulators through band structure combinatorics. Phys. Rev. X.

[57] Po HC, Vishwanath A, Watanabe H (2017). Complete theory of symmetry-based indicators of band topology. Nat. Commun..

[58] Zak J (1982). Band representations of space groups. Phys. Rev. B.

[59] Serbyn M, Fu L (2014). Symmetry breaking and landau quantization in topological crystalline insulators. Phys. Rev. B.

[60] Bouhon A, Black-Schaffer AM, Slager R-J (2019). Wilson loop approach to fragile topology of split elementary band representations and topological crystalline insulators with time-reversal symmetry. Phys. Rev. B.

[61] Kooi SH, van Miert G, Ortix C (2019). Classification of crystalline insulators without symmetry indicators: Atomic and fragile topological phases in twofold rotation symmetric systems. Phys. Rev. B.

[62] Hwang Y, Ahn J, Yang B-J (2019). Fragile topology protected by inversion symmetry: Diagnosis, bulk-boundary correspondence, and Wilson loop. Phys. Rev. B.

[63] Song Z-D, Elcoro L, Xu Y-F, Regnault N, Bernevig BA (2020). Fragile phases as affine monoids: Classification and material examples. Phys. Rev. X.

[64] Song Z-D, Elcoro L, Bernevig BA (2020). Twisted bulk-boundary correspondence of fragile topology. Science.

[65] Peri V, Song Z-D, Serra-Garcia M, Engeler P, Queiroz R, Huang X, Deng W, Liu Z, Bernevig BA, Huber SD (2020). Experimental characterization of fragile topology in an acoustic metamaterial. Science.

[66] Song Z, Zhang T, Fang Z, Fang C (2018). Quantitative mappings between symmetry and topology in solids. Nat. Commun..

[67] Zhou X, Hsu C-H, Chang T-R, Tien H-J, Ma Q, Jarillo-Herrero P, Gedik N, Bansil A, Pereira VM, Xu S-Y, Lin H, Fu L (2018). Topological crystalline insulator states in the Ca_2_As family. Phys. Rev. B.

[68] Hsu C-H , Zhou X, Ma Q, Gedik N, Bansil A, Pereira VM, Lin H, Fu L, Xu S-Y, Chang T-R (2019). Purely rotational symmetry-protected topological crystalline insulator-Bi_4_Br_4_. 2D Mater..

[69] Haldane FDM (1988). Model for a quantum hall effect without landau levels: Condensed-matter realization of the “parity anomaly”. Phys. Rev. Lett..

[70] Hatsugai Y, Kohmoto M, Wu Y-S (1996). Hidden massive Dirac fermions in effective field theory for integral quantum hall transitions. Phys. Rev. B.

[71] Watanabe H, Hatsugai Y, Aoki H (2010). Half-integer contributions to the quantum hall conductivity from single Dirac cones. Phys. Rev. B.

[72] Jain A, Ong SP, Hautier G, Chen W, Richards WD, Dacek S, Cholia S, Gunter D, Skinner D, Ceder G, Persson KS (2013). The Materials Project: A materials genome approach to accelerating materials innovation. APL Mater..

[73] Jaiganesh G, Britto TM, Eithiraj RD, Kalpana G (2008). Electronic and structural properties of NaZnx (x= p, as, sb): an ab initio study. J. Phys. Condes. Matter.

[74] Savelsberg G, Schaefer H (1978). Ternaere pnictide und chalkogenide von alkalimetallen und IB-bzw. IIB-elementen. Z. Naturforsch. B.

[75] Kahlert H, Schuster H-U (1976). Ternary phases of sodium or potassium with elements of the 2 b- and 5 b-group. Z. Naturforsch. B.

[76] Nordheim L (1931). Zur elektronentheorie der metalle. I. Ann. Phys..

[77] Bellaiche L, Vanderbilt D (2000). Virtual crystal approximation revisited: Application to dielectric and piezoelectric properties of perovskites. Phys. Rev. B.

[78] Alexandradinata A, Dai X, Bernevig BA (2014). Wilson-loop characterization of inversion-symmetric topological insulators. Phys. Rev. B.

[79] Alexandradinata A, Bernevig BA (2016). Berry-phase description of topological crystalline insulators. Phys. Rev. B.

[80] Alexandradinata A, Wang Z, Bernevig BA (2016). Topological insulators from group cohomology. Phys. Rev. X.

[81] Lee H, Kang Y-G, Jung M-C, Han MJ, Chang KJ (2022). Robust dual topological insulator phase in NaZnBi. NPG Asia Mater..

[82] Khalaf E, Po HC, Vishwanath A, Watanabe H (2018). Symmetry indicators and anomalous surface states of topological crystalline insulators. Phys. Rev. X.

[83] Giannozzi P (2009). Quantum espresso: A modular and open-source software project for quantum simulations of materials. J. Phys. Condens. Matter.

[84] Perdew JP, Burke K, Ernzerhof M (1996). Generalized gradient approximation made simple. Phys. Rev. Lett..

[85] Rappe AM, Rabe KM, Kaxiras E, Joannopoulos JD (1990). Optimized pseudopotentials. Phys. Rev. B.

[86] Ramer NJ, Rappe AM (1999). Designed nonlocal pseudopotentials for enhanced transferability. Phys. Rev. B.

[87] Monkhorst HJ, Pack JD (1976). Special points for Brillouin-zone integrations. Phys. Rev. B.

[88] Soluyanov AA, Vanderbilt D (2011). Wannier representation of z_2_ topological insulators. Phys. Rev. B.

[89] Marzari N, Vanderbilt D (1997). Maximally localized generalized Wannier functions for composite energy bands. Phys. Rev. B.

[90] Souza I, Marzari N, Vanderbilt D (2001). Maximally localized Wannier functions for entangled energy bands. Phys. Rev. B.

[91] Mostofi AA, Yates JR, Lee Y-S, Souza I, Vanderbilt D, Marzari N (2008). wannier90: A tool for obtaining maximally-localised Wannier functions. Comput. Phys. Commun..

[92] Taherinejad M, Garrity KF, Vanderbilt D (2014). Wannier center sheets in topological insulators. Phys. Rev. B.

[93] Marzari N, Mostofi AA, Yates JR, Souza I, Vanderbilt D (2012). Maximally localized Wannier functions: Theory and applications. Rev. Mod. Phys..

