# Supplementary material for: Topological phase transitions without symmetry indication in NaZnSb1-xBix
Source: Sci Rep. 2022 Dec 21;12:22050. doi: 10.1038/s41598-022-26596-y (PMC9772356; doi:10.1038/s41598-022-26596-y)
Supplement: Supplementary file 1 — Supplementary Information. [file 41598_2022_26596_MOESM1_ESM.pdf]

Supplemental Materials for "Topological phase transitions without symmetry indication in $NaZnSb_{1-x}Bi_x$"

Jaemo Jeong,[1] Dongwook Kim,[2] and Youngkuk Kim[1,*]

[1] *Department of Physics, Sungkyunkwan University, Suwon 16419, Korea*

[2] *Department of Materials Science and Engineering, University of Utah, Salt Lake City, Utah 84112, USA*

(Dated: June 23, 2022)

WILSON LOOP SPECTRA

Figure S1(a) shows that the band gap calculated from the DFT bands as a function of $x \in [0, 1]$. The corresponding topological invariants are displayed, labeled by topological invariants $(\mu_x, \mu_{xy}\nu_0)$. The topological phases are identified by calculating the mirror-specified Wilson loop calculations. The results are shown in Figs. S1(b)-S1(e).

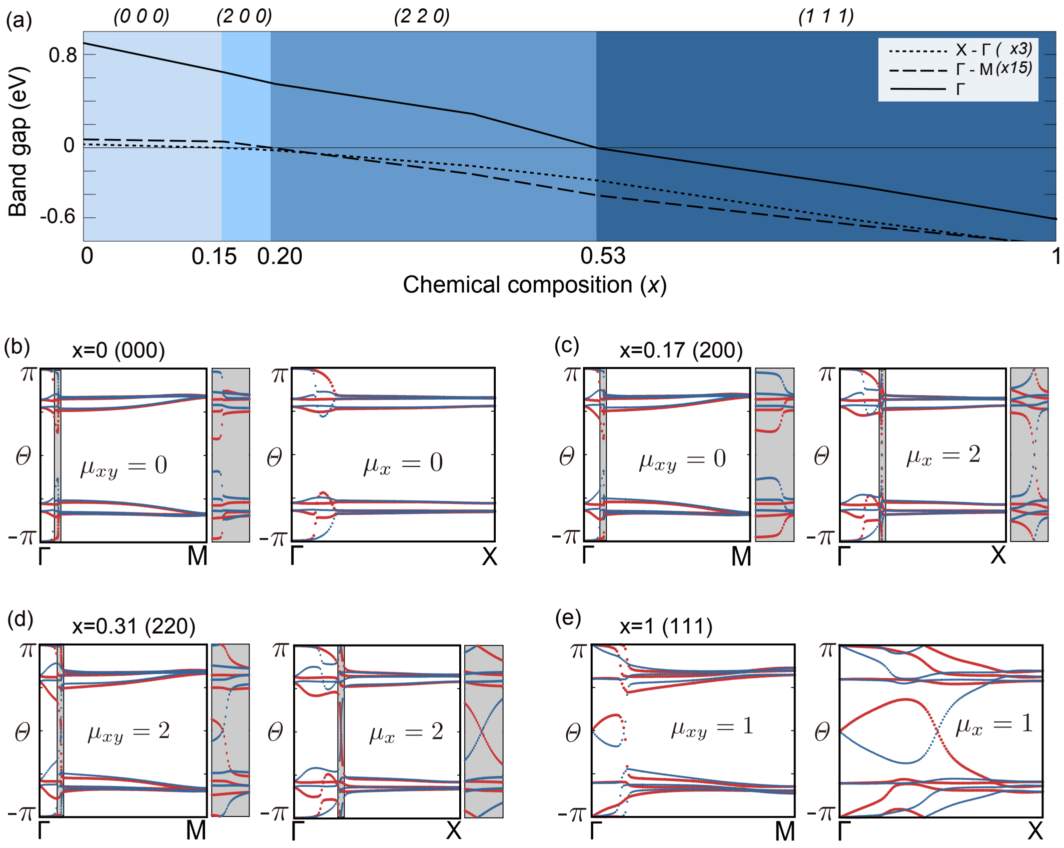

FIG. S1. (a) Topological phase diagram of $NzZnSb_{1-x}Bi_x$ in chemical composition x-space. The energy bandgap in the high-symmetry lines $X-\Gamma$, $\Gamma-M$, and at Γ are shown as a function of x. The solid dashed and dotted lines correspond to the gaps of Γ,$\Gamma-M$, and $X-\Gamma$, respectively. Negative values are assigned to the bandgap when the gaps are reopened after the occurrence of the (zero-gap) Dirac point. The bandgap of the $X-\Gamma$ line is multiplied by three times. The bandgap of the $\Gamma-M$ line is fifteen times multiplied. The computed topological indices $(\mu_x\mu_{xy}\nu_0)$ are presented in the corresponding domains, where μ_x and μ_{xy} are two mirror Chern numbers and ν_0 is the strong $\mathbb{Z}_2$ topological invariant. Different color schemes are used to represent other topological domains. (b)-(e) Mirror-specific Wilson loop spectra of $NaZnSb_xBi_{1-x}$ for various chemical compositions x. The grey boxes are magnified in the right panel. (b) x = 0: triival phase with $(\mu_x\mu_{xy}\nu_0)$ = (000). (c) x = 0.17: topological cyrstalline phase with $(\mu_x\mu_{xy}\nu_0)$ = (200). (d) x = 0.31: topological cyrstalline phase with $(\mu_x\mu_{xy}\nu_0)$ = (220). (e) x = 1: strong topological insulator phase with $(\mu_x\mu_{xy}\nu_0)$ = (111). Blue and red colors indicate the bands with the $+i$ and -i eigenvalues, respectively.

$\boldsymbol{k}\cdot\boldsymbol{p}$ MODEL

Eight-band Model

This section develops a low-energy effective theory that reproduces the DFT bands near the Γ point. We first specify the representations of the symmetry operators in the space group #129 ($P4/nmm$). The crystal structure of tetragonal matlockite-type $\mathrm{NaZnSb}_x\mathrm{Bi}_{1-x}$ belongs to the nonsymmorphic space group #129 ($P4/nmm$). The corresponding little group of Γ is D_{4h} ($4/mmm$). The point group D_{4h} ($4/mmm$) can be generated by inversion $\mathcal{P}$, and two rotations $\mathcal{C}_{2x}$ and $\mathcal{C}_{4z}$. We are interested in the effective model that describes the (N_e-3)-th to (N_e+4)-th DFT bands in the vicinity of Γ, where N_e is the number of electrons provided from a single unit cell. The DFT calculations show that the representations for these Bloch states at Γ are Γ^{7-}, Γ^{6+}, Γ^{6-}, and Γ^{7-}, from $(N-3)$-th to $(N+4)$-th states, respectively. Using them as a basis set $\{|\Gamma^{7-},\pm\rangle, |\Gamma^{6+},\pm\rangle, |\Gamma^{6-},\pm\rangle, |\Gamma^{7-},\pm\rangle\}$ the symmetry representations are found as $\mathcal{T} = i\sigma_y K$, P $=\{(1+\mu_z)\tau_z + (1-\mu_z)\}/2$, $C_{2x} = i\mu_z\tau_z(\sigma_x+\sigma_y)/\sqrt{2}$, $C_{4z} = \mu_z\tau_z e^{-i\pi\sigma_z/4}$, where μ_i and τ_i (σ_i) are the Pauli matrices describing the orbital (spin) degree of freedoms and K is complex conjugate operator. In a matrix form,

$$\mathcal{P} = \begin{pmatrix} 1 & 0 & 0 & 0 \\ 0 & -1 & 0 & 0 \\ 0 & 0 & 1 & 0 \\ 0 & 0 & 0 & 1 \end{pmatrix}, \mathcal{C}_{2x} = \begin{pmatrix} 1 & 0 & 0 & 0 \\ 0 & -1 & 0 & 0 \\ 0 & 0 & -1 & 0 \\ 0 & 0 & 0 & 1 \end{pmatrix} \otimes \frac{\sigma_x+\sigma_y}{\sqrt{2}} i, \mathcal{C}_{4z} = \begin{pmatrix} 1 & 0 & 0 & 0 \\ 0 & -1 & 0 & 0 \\ 0 & 0 & -1 & 0 \\ 0 & 0 & 0 & 1 \end{pmatrix} \otimes \left(e^{\frac{-\sigma_z \pi i}{4}}\right). \tag{S1}$$

As a result, the mirror operations M_y and $M_{x\bar{y}}$ are represented as

$$M_y = \begin{pmatrix} 1 & 0 & 0 & 0 \\ 0 & 1 & 0 & 0 \\ 0 & 0 & -1 & 0 \\ 0 & 0 & 0 & 1 \end{pmatrix} \otimes \left(\frac{\sigma_x-\sigma_y}{\sqrt{2}} i\right), M_{x\bar{y}} = \begin{pmatrix} -1 & 0 & 0 & 0 \\ 0 & 1 & 0 & 0 \\ 0 & 0 & -1 & 0 \\ 0 & 0 & 0 & -1 \end{pmatrix} \otimes (\sigma_x i) \tag{S2}$$

In terms of the relative momenta $\boldsymbol{q} \equiv \boldsymbol{k} - \Gamma$, the 8×8 effective Hamiltonian can be obtained up to the cubic order by imposeing the symmetric constraint

$$\mathcal{H}(\hat{O}_g \boldsymbol{k}) = U_g^\dagger \mathcal{H}(\boldsymbol{q}) U_g, \tag{S3}$$

where U_g is the representation for the generator g of the point group D_{4h} ($4/mmm$), and $\hat{O}_g$ is corresponding $SO(3)$ representation in the momentum space. The effective Hamiltonian is given by

$$
\begin{aligned}
\mathcal{H}(\boldsymbol{k}) = & \sum_{\xi\in\Xi_1} \xi\Theta_\xi^{\text{even}} + \sum_{\xi\in\Xi_2} \xi\left[\sigma_x\Theta_\xi^{\text{odd}+} + \sigma_x\Theta_\xi^{\text{odd}-}\right]\Theta_\xi^{\text{even}} \\
& + \left[m_1\mu_x(1+\tau_z) + m_2(1-\mu_x)\tau_x\right]k_xk_y \\
& + m_3\mu_x(\tau_x - \tau_z)\sigma_z k_xk_yk_z \\
& + m_4(\mu_x\tau_x + \mu_y\tau_y)\sigma_z k_z,
\end{aligned} \tag{S4}
$$

where Ξ_1 is a 8×8 matrix vector $\Xi_1 = \{1, \mu_z, \tau_z, \mu_z\tau_z, \mu_x\tau_x - \mu_z\tau_z\}$ and Ξ_2 is 4×4 matrix vector $\Xi_2 = \{\mu_y\tau_y + \mu_x\tau_x, \mu_x(1-\tau_z), (1-\mu_z)\tau_x, (\mu_x\tau_y + \mu_y\tau_x)k_z, \mu_y(1+\tau_z)k_z, (1-\mu_z)\tau_yk_z\}$, and polynomial funtions of $\boldsymbol{q}$ are denoted as $\Theta_\xi^{\text{even}} = a_\xi + b_\xi(k_x^2+k_y^2) + c_\xi k_z^2$, $\Theta_\xi^{\text{odd}+} = (k_x+k_y) + d_\xi(k_x^3+k_y^3)$, and $\Theta_\xi^{\text{odd}-} = (k_x - k_y) + e_\xi(k_x^3 - k_y^3)$.

$\{\Gamma^{6+}, \Gamma^{6-}\}$ Four-band Model

Using the basis $\{|\Gamma^{6+}, \pm\rangle, |\Gamma^{6-}, \pm\rangle\}$. The symmetry gnerators are represented as

$$
\mathcal{P} = \begin{pmatrix} -1 & 0 \\ 0 & 1 \end{pmatrix}; \mathcal{C}_{2x} = \begin{pmatrix} -1 & 0 \\ 0 & -1 \end{pmatrix} \otimes \frac{\sigma_x + \sigma_y}{\sqrt{2}} i; \mathcal{C}_{4z} = \begin{pmatrix} -1 & 0 \\ 0 & -1 \end{pmatrix} \otimes e^{\frac{-\sigma_z \pi i}{4}}. \tag{S5}
$$

Similarily, mirrors are given by

$$
\mu_y = i\begin{pmatrix} 1 & 0 \\ 0 & -1 \end{pmatrix} \otimes \frac{\sigma_x - \sigma_y}{\sqrt{2}}; \mu_{x\overline{y}} = i\begin{pmatrix} 1 & 0 \\ 0 & -1 \end{pmatrix} \otimes \sigma_x. \tag{S6}
$$

The effective Hamiltonian for the Γ^{6+} and Γ^{6-} states can be found as

$$
\mathcal{H}(k_x, k_y, k_z) = \begin{pmatrix} A & 0 & E & C-D \\ 0 & A & C+D & -E \\ E & C-D & B & 0 \\ C+D & -E & 0 & B \end{pmatrix}. \tag{S7}
$$

Here, $A = a_0 + a_1(k_x^2 + k_y^2) + a_2 k_z^2$, $B = b_0 + b_1(k_x^2 + k_y^2) + b_2 k_z^2$, $C = c_0(k_x + k_y)k_z$, $D = d_0(k_x - k_y)k_z$, and $E = e_0 k_z$. When $x_c = 0.60625$ and $k_x = k_y = k_z = 0$, the Dirac point appears at the Γ point, describing the topological phase transition dictated by ν_0.

$\{\Gamma^{7-}, \Gamma^{7-}\}$ Four-band Model

We distill the four-band model to describe the $\{|\Gamma^{7-}, \pm\rangle, |\Gamma^{7-}, \pm\rangle\}$ states. The symmetry operators are represented as below:

$$\mathcal{P} = \begin{pmatrix} 1 & 0 \\ 0 & 1 \end{pmatrix}; \mathcal{C}_{2x} = \begin{pmatrix} 1 & 0 \\ 0 & 1 \end{pmatrix} \otimes \frac{\sigma_x + \sigma_y}{\sqrt{2}} i; \mathcal{C}_{4z} = \begin{pmatrix} 1 & 0 \\ 0 & 1 \end{pmatrix} \otimes \left(e^{\frac{-\sigma_z \pi i}{4}} \right). \tag{S8}$$

Similarly, for the mirror symmetries:

$$\mu_y = i \begin{pmatrix} 1 & 0 \\ 0 & 1 \end{pmatrix} \otimes \frac{\sigma_x - \sigma_y}{\sqrt{2}}; \mu_{x\bar{y}} = i \begin{pmatrix} -1 & 0 \\ 0 & -1 \end{pmatrix} \otimes \sigma_x. \tag{S9}$$

The effective Hamiltonian for Γ^{7-} and Γ^{7-}:

$$\mathcal{H}(k_x, k_y, k_z) = \begin{pmatrix} A & 0 & E & -C - D \\ 0 & A & C - D & E \\ E & C + D & B & 0 \\ -C + D & E & 0 & B \end{pmatrix}. \tag{S10}$$

Here, $A = a_0 + a_1(k_x^2 + k_y^2) + a_2 k_z^2$, $B = b_0 + b_1(k_x^2 + k_y^2) + b_2 k_z^2$, $C = c_0(k_x - k_y)k_z$, $D = d_0(k_x + k_y)k_z$, and $E = e_0 + e_1(k_x^2 + k_y^2) + e_2 k_z^2$. The Dirac points occur at $x = 0.145$ off Γ, describing the mirror Chern number change.

Mirror Chern Number on the $k_x = 0$ Plane

Here, we calculate the mirror Chern number μ_x on the $k_x = 0$ plane from the $\{\Gamma^{7-}, \Gamma^{7-}\}$ Hamiltonain:

$$\mathcal{H}(k_y, k_z) = \begin{pmatrix} A & 0 & B & (-1-i)C \\ 0 & A & (1-i)C & B \\ B & (1+i)C & -A & 0 \\ (-1+i)C & B & 0 & -A \end{pmatrix} \tag{S11}$$

where $A \equiv a_0 + a_1 k_y^2 + a_2 k_z^2$, $B \equiv b_0 + b_1 k_y^2 + b_2 k_z^2$, and $C \equiv c_2 k_y k_z \tau_z$, and $a_0, a_1, a_2, b_0, b_1, b_2$, and c_2 are parameters. We can rewrite the Hamiltonian as Pauli matrix. $\{\tau, \sigma\}$ represent orbital and spin part.

$$\mathcal{H}(k_y, k_z) = (a_0 + a_1 k_y^2 + a_2 k_z^2)\tau_z \tag{S12}$$

$$+ (b_0 + b_1 k_y^2 + b_2 k_z^2)\tau_x \tag{S13}$$

$$+ c_2 k_y k_z (\sigma_x + \sigma_y)\tau_y. \tag{S14}$$

The mirror symmetry operator

$$\frac{i}{\sqrt{2}}(\sigma_x + \sigma_y) \tag{S15}$$

can be diagonalized into

$$i\tilde{\sigma}_z = \frac{i}{\sqrt{2}}(\sigma_x + \sigma_y), \tag{S16}$$

where $\sigma_z = \pm 1$. In this basis, the Hamiltonian Eq. S12 is block-diagonalized as

$$\mathcal{H}(k_y, k_z) = (a_0 + a_1 k_y^2 + a_2 k_z^2)\tau_z \tag{S17}$$

$$+ (b_0 + b_1 k_y^2 + b_2 k_z^2)\tau_x \tag{S18}$$

$$+ \tilde{c}_2 k_y k_z \tau_y \tilde{\sigma}_z, \tag{S19}$$

where $\tilde{c} = \sqrt{2}c$. Note that the last term describes spin-orbit coupling. The corresponding eigenenergies are

$$E_{\pm}^2 = (a_0 + a_1 k_y^2 + a_2 k_z^2)^2 \tag{S20}$$

$$+ (b_0 + b_1 k_y^2 + b_2 k_z^2)^2 \tag{S21}$$

$$+ (\tilde{c}_2 k_y k_z)^2$$

$$= A(k_y, k_z)^2 + B(k_y, k_z)^2 + \tilde{C}(k_y, k_z)^2, \tag{S22}$$

where $\tilde{C}(k_y, k_z) \equiv \tilde{c}_2 k_y k_z \tilde{\tau}_z$. Depending on the parameters a_i, b_i, c_2 $(i = 0, 1, 2)$, $A = B = \tilde{C} = 0$ can occur, allowing for band crossing $E_+ = E_-$. For the case of $NaZnSb_{1-x}Bi_x$, our first-principles calculations show that $a_0 a_2 > 0$, $a_0 a_1 < 0$, and $\tilde{c}_2 \neq 0$. Therefore, nodal points can occur only at $k_y^2 = k_0^2 \equiv -a_0/a_1$ and $k_z = 0$ when

$$\frac{a_0}{a_1} = \frac{b_0}{b_1}. \tag{S23}$$

This condition defines the parameter set tuned to a topological critical point, where the mirror Chern number μ_x changes. Using the method proposed in Ref. [**?**], the mirror Chern number μ_x is calculated as

$$\mu_x = \mathrm{sgn}\left[\tilde{c}_2 b_0 \left(a_0 - a_1 \frac{b_0}{b_2}\right)\right] - \mathrm{sgn}\left[\tilde{c}_2 b_0 \left(a_0 - a_1 \frac{b_0}{b_1}\right)\right], \tag{S24}$$

or equivalently,

$$\mu_x = \mathrm{sgn}\left[\tilde{c}_2 \left(\frac{a_0}{b_0} - \frac{a_2}{b_2}\right)\right] - \mathrm{sgn}\left[\tilde{c}_2 \left(\frac{a_0}{b_0} - \frac{a_1}{b_1}\right)\right]. \tag{S25}$$

The second term changes its sign from + to - at Eq. S23 as the chemical composition x increases. Consequently, the mirror Chern number $\mu_x = 2$ on the $k_x = 0$ plane changes from 0 to 2, inducing a nontrivial topological crystalline insulator phase.

Chern Number Formula

Here we derive the mirror Chern number formula (Eq. S25). Let us consider a general two-band Hamiltonian on two-dimensional momentum space (k_x, k_y)

$$\mathcal{H}(k_x k_y) = \sum_{i=0,1,2,3} h_i(k_x, k_y)\sigma_i \tag{S26}$$

σ_i with $(i = 0, 1, 2, 3)$ is the Pauli matrix and $h_i(\boldsymbol{k})$ is a momentum $\boldsymbol{k}$-dependent parameter. The eigenenergy of $\mathcal{H}(\boldsymbol{k})$ is $E_\pm(\boldsymbol{k}) = \pm\sqrt{h_1(\boldsymbol{k})^2 + h_2(\boldsymbol{k})^2 + h_3(\boldsymbol{k})^2}$. The Dirac point occurs if and only if $h_1 = h_2 = h_3 = 0$. As derived in Ref. [**?**], the Chern number formula can be represented as a function of h_i. By considering h_3 as a mass term, one can arrive at

$$\mathcal{C} = \frac{1}{2}\sum_{\boldsymbol{k}_\mathrm{D}} \mathrm{sgn}(\partial_{k_x}\boldsymbol{h} \times \partial_{k_y}\boldsymbol{h})_3 \mathrm{sgn}(h_3), \tag{S27}$$

where $\boldsymbol{h} = (h_1, h_2, h_3)$ and $\{\boldsymbol{k}_\mathrm{D}\}$ corresponds to the solutions of $h_1 = h_2 = 0$. The mirror specific Chern number

$$\mu_z = \frac{1}{2}(\mathcal{C}_{+i} - \mathcal{C}_{-i})$$

can be calculated by evaluating the mirror-specific Chern number $\mathcal{C}_{\pm i}$ from the block-diagonalized Hamiltonian $\mathcal{H} = \mathcal{H}_{+i} \oplus \mathcal{H}_{-i}$. In particular, fhe $\{\Gamma^{7-}, \Gamma^{7-}\}$ four-band Hamiltonian (Eq. S11) can be block-diagonalized using $M_{x\bar{y}}$ operator under the mirror-invariant plane $k_x = k_y$ where $h_1 \equiv a_0 + a_1 k_\parallel^2 + a_2 k_z^2$, $h_2 \equiv b_0 + b_1 k_\parallel^2 + b_2 k_z^2$, $h_3 \equiv c_2 k_\parallel k_z$, and $k_\parallel^2 \equiv k_x^2 + k_y^2$.

$$\mathcal{H}_{\pm i}(k_\parallel, k_z) = h_1(k_\parallel, k_z)\sigma_2 + h_2(k_\parallel, k_z)\sigma_1 \pm h_3(k_\parallel, k_z) \tag{S28}$$

Consequently, the mirror specific Chern number $\mu_{x\bar{y}}$ becomes

$$\mu_{x\bar{y}} = \mathrm{sgn}\left[c_2\left(\frac{a_0}{b_0} - \frac{a_2}{b_2}\right)\right] - \mathrm{sgn}\left[c_2\left(\frac{a_0}{b_0} - \frac{a_1}{b_1}\right)\right]. \tag{S29}$$
